# Oral Mucosal Lesions in Indians From Northeast Brazil: Cross-Sectional Study of Prevalence and Risk Indicators

**DOI:** 10.1097/MD.0000000000000140

**Published:** 2014-12-12

**Authors:** Patricia Ramos Cury, Lia Pontes Arruda Porto, Jean Nunes dos Santos, Livia Silva Figueiredo e Ribeiro, Flavia Caló de Aquino Xavier, Andreia Leal Figueiredo, Luciana Maria Pedreira Ramalho

**Affiliations:** From the Department of Periodontics (PR); Postgraduate Program in Health and Dentistry (LPAP, LSFER); Department of Oral Pathology (JNDS, FCDAX, LMPR); and Department of Social Dentistry, School of Dentistry, Federal University of Bahia, Salvador, Bahia, Brazil (ALF).

## Abstract

The aim of this cross-sectional study was to evaluate the prevalence of oral mucosal lesions, and their risk indicators in adult Kiriri Indians from Northeast Brazil.

Clinical oral examination was performed on a representative sample of 223 Indians (age ≥19 years). A systematic evaluation of lips, labial mucosa and sulcus, commissures, buccal mucosa and sulcus, gingiva and alveolar ridge, tongue, floor of the mouth, and soft and hard palate was performed. Bivariate analysis was conducted to assess associations between mucosal conditions and age, gender, income, educational level, diabetic status, and smoking status.

Mucosal lesions were found in 50 participants (22.4%). The most prevalent lesions were fistulae (6.2%) and traumatic ulcers (4.48%). Oral mucosal was associated with higher age (≥35 years; odds ratio [OR] = 1.99, 95% confidence interval [CI]: 1.05–3.76, *P* = 0.03) and lower education level (<9 years; OR = 2.13, 95% CI: 0.96–4.71, *P* = 0.06).

Mucosal conditions are prevalent in Kiriri Indians and the presence of mucosal lesions is associated with advanced age and lower education. A public health program aimed at preventing and treating mucosal lesions and targeted toward the high-risk group is vital to improve the oral health status of this population.

## INTRODUCTION

Prevalence of oral mucosal lesions (OMLs) varies between different populations. Prevalence of OMLs between 9.7% and 61.6% has been reported in adults.^1–4^ Therefore, epidemiological investigations play an essential role in determining the treatment needs of populations and in tailoring oral health programs that are specific to each population.

Some Indian communities are largely isolated from external influences and have characteristic gene pools, environmental exposures, cultures, and traditions, all of which make them unique. This may result in differences in the prevalence of OMLs and their risk factors. Epidemiological studies of oral diseases in these populations are scarce owing to logistical, financial, and political constraints. A few studies have evaluated OMLs in Navajo Indians from USA, and in Kaigang, Waimiri Atroari, Umutina, Paresi, Bororo, Bakairi, Kayabi, Irantxe, Nambikwara, and Terena Indians from Brazil.^5–8^ Prevalence of between 4.6% and 44.9% has been reported.^6–8^

Brazil has a total population of 190,732,694, including more than 817,000 Indians,^9^ who account for 0.4% of the population. They live in urban areas or in 688 Indian communities.^10^ The Kiriri Indians live isolated in the northern region of Bahia state in Northeast Brazil. The Kiriri Indian land covers an area of 12,300 hectares and has a semiarid climate. The area is remote and difficult to access, and external influence is limited. The Indian community in this study is a population of approximately 2182 individuals. Although this population has had some external contact, they still maintain their social, cultural, behavioral, and genetic background. Among this population, cultural beliefs, health care traditions, difficulties in accessing health care, and high costs are all factors that inhibit access to dental services. Their nutrition is based on beans, manioc, and corn. Consumption of sugar added to coffee and high-sugar foods is frequent. Sweet potato, pumpkin, okra, tomato, lettuce, and tropical fruits are part of the nutrition, whereas meat, milk, and milk derivatives are not available to all. To the best of our knowledge, no previous epidemiological study of the mucosal conditions in this population has been performed. It has been shown that periodontitis is highly prevalent in Kiriri Indians, but only few teeth show advanced disease, and periodontitis was associated with higher age, male sex, and diabetes.^11^ The aim of this study was to characterize the prevalence of OMLs in adult Kiriri Indians from Northeast Brazil and to determine the association between these conditions and age, gender, income, educational level, diabetic status, and smoking status.

## METHODS

This study was conducted in accordance with the World Medical Association Declaration of Helsinki. It was approved by the Brazilian Research Ethics Committee of the Ministry of Health, Brasília, Brazil, and by Indian authorities and the Brazilian National Health Foundation (FUNASA). All participants provided written, informed consent. At the conclusion of the study, the Indian authorities and FUNASA were provided with a written report of the study results, and each participant was informed about their oral health status. Individuals with OMLs were referred to the stomatology service of the Federal University of Bahia for biopsies, when indicated.

### Study Design and Sampling Procedures

A cross-sectional observational analytic study design was used. The target population comprised Kiriri Indians age ≥19 years who were living in an isolated Indian area in the state of Bahia in Northeast Brazil.

A representative sample of adults was calculated based on information provided by FUNASA. Of the 2182 Kiriri Indians living in the isolated Kiriri Indian area in 2011, 1025 were adults (age ≥19 years). A sampling error of 5%, confidence level of 95%, and maximum percentage of OMLs of 62% were considered in the sample size calculation. The calculated required sample size was 268 persons. The persons were randomly selected from the list of 1025 adults provided by FUNASA. The local Indians authorities first invited the community to participate. A few days before the clinical examinations, the community dentist and 2 nurses visited the selected participants to explain the aims of the study and encourage participation. The response rate was 84.3%.

Two hundred twenty-six individuals ranging in age from 19 to 77 years were examined. Three participants with missing data on oral mucosal conditions were excluded from the statistical analysis.

### Operational Procedure in the Field

The fieldwork was performed during 2011. Before clinical examination, face-to-face interviews were conducted by 2 trained nurses to obtain background data on demographic and socioeconomic status as well as other health-related data using a structured written questionnaire. A fasting glucose blood test was performed on all participants (OneTouch Ultra Mini, Lifescan, Milpitas, CA).

### Oral Mucosa Evaluation

Four trained examiners performed the clinical examinations and were assisted by 4 trained undergraduate students from the School of Dentistry of the Federal University of Bahia.

Clinical examination was performed using a headlight (Turboled, Nautika, São Paulo, São Paulo, Brazil), with the participants seated in a regular chair in 10 rudimentary sites, including schools and health facilities. Biosafety standards were properly followed using sterilized gauze, plane dental mirror, gloves, and biohazard protection material. Examination of the oral mucosa was performed according to World Health Organization criteria.^12^ Clinical examination procedures included the systematic evaluation of lips, labial mucosa and sulcus, commissures, buccal mucosa and sulcus, gingiva and alveolar ridge, tongue, floor of the mouth, and soft and hard palate. Color, texture, and any abnormalities were evaluated. When an oral mucosal condition was found, the location, clinical diagnosis, and clinical description were registered.

Diagnosis of oral mucosal conditions was performed following a stepwise protocol including clinical examination by the field examiners, review of clinical records and photos by 1 experienced pathologist, and referral of cases to the stomatology service of the Federal University of Bahia for further clinical examination, treatment and biopsies whenever necessary.

### Data Analysis

The statistical analysis included 223 participants. Prevalence of OMLs and variations of normality were calculated. Data analysis was performed using SPSS version 13.0 (SPSS Inc., Chicago, IL).

Demographic and socioeconomic status and other health-related data were categorized. Age was divided into 2 categories: 19 to 34 and ≥35 years. Educational level was categorized as ≥9 years of education or <9 years of education. Economic status was categorized as monthly income ≥US$259 or <US$259. Participants were classified as current smokers or nonsmokers. If the participants had a self-reported physician's diagnosis of diabetes or fasting blood glucose level ≥126 mg/dL associated with diabetes symptoms (increased thirst, increased urination, and unexplained weight loss), they were considered to have diabetes. Otherwise, they were considered not to have diabetes.

Descriptive statistics of oral mucosal conditions according to age, gender, education, income, smoking status, and diabetes status were performed. A chi-square test was used to compare participants with and without oral mucosal alterations, according to the aforementioned variables. The odds ratios (OR) together with 95% confidence intervals (CI) were calculated. *P* values of <0.05 were considered significant.

## RESULTS

Forty-four percent (44.8%) of the participants were men. Most participants (60.9%) were ≤35 years, had <9 years of education (66.8%), and had an income of less than US$259 per month (80.3%). Only 14 participants had diabetes (6.3%). Approximately, 50% (50.7%) were current smokers, and the mean (standard deviation [SD]) number of cigarettes, cigars, or pipes smoked per day was 3.7 (3.8).

OMLs were found in 50 Indians (22.4%), 58% in women, and 52% in individuals age ≥35 years (Table 1). Ten individuals had >1 lesion. Sixteen different types of lesion were diagnosed clinically. The most prevalent type of lesion was fistulae (6.2%), followed by traumatic ulcers (4.4%) and melanocytic nevi (2.6%). The other types of lesion diagnosed are shown in Table 2. The most commonly affected areas were the lips, gingiva, and buccal mucosa (30.1%, 28.5% and 19.0%, respectively).

**TABLE 1:**
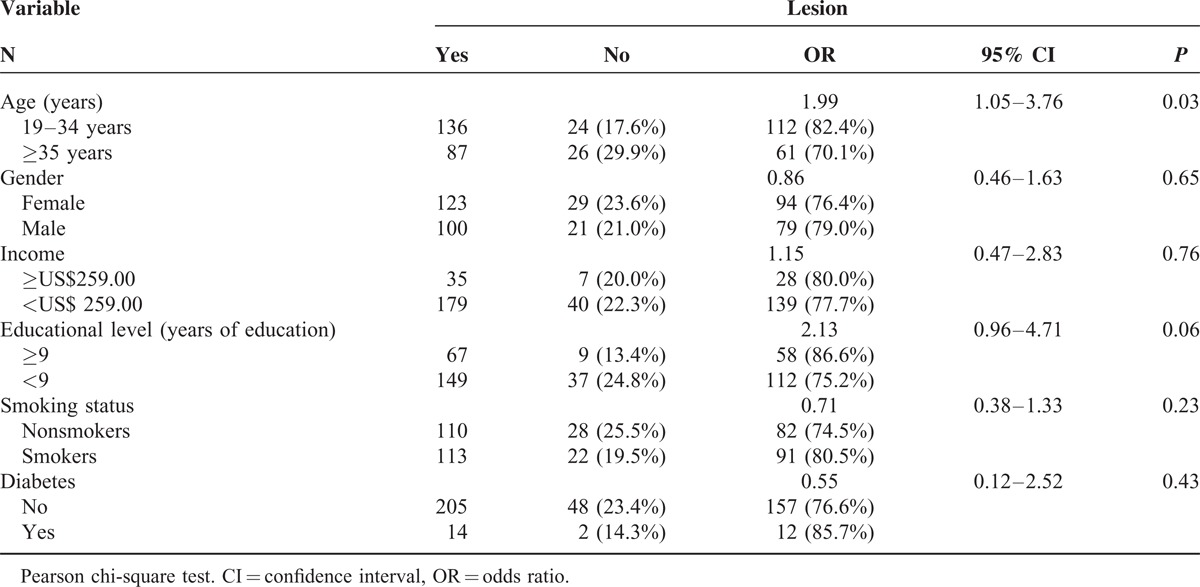
Bivariate Analysis of the Association Between Age, Gender, Income, Educational Level, Smoking Status and Diabetes and the Occurrence of Oral Mucosal Lesions (n = 223), State of Bahia, Brazil, 2011

**TABLE 2:**
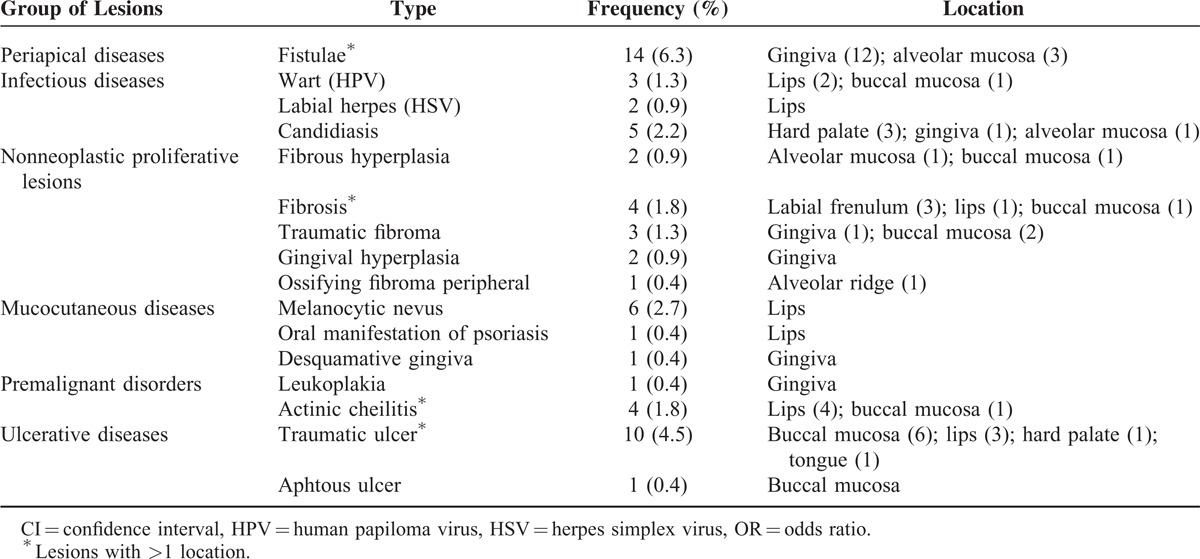
Prevalence of Oral Mucosal Lesions Among Adult Kiriri Indians (n = 223), State of Bahia, Brazil, 2011

Bivariate analysis showed that participants age ≥35 years were at a significantly higher risk of having OMLs than younger participants (OR: 1.99, 95% CI: 1.05–3.76, *P* = 0.03) (Table 1). There was a tendency toward higher risk of OMLs in less-educated individuals (OR: 2.13, 95%CI: 0.96–4.71, *P* = 0.06). Gender, income, smoking status, and diabetes status were not significantly associated with OMLs.

Variations from normal mucosa were identified in 144 (64.5%) participants. Fourteen participants had >1 alteration. The most prevalent variations were melanin pigmentation (57.3%) and fissured tongue (7.6%) (Table 3). Age, gender, income, education level, smoking status, and diabetes status were not significantly associated with variations of normality (Table 4).

**TABLE 3:**
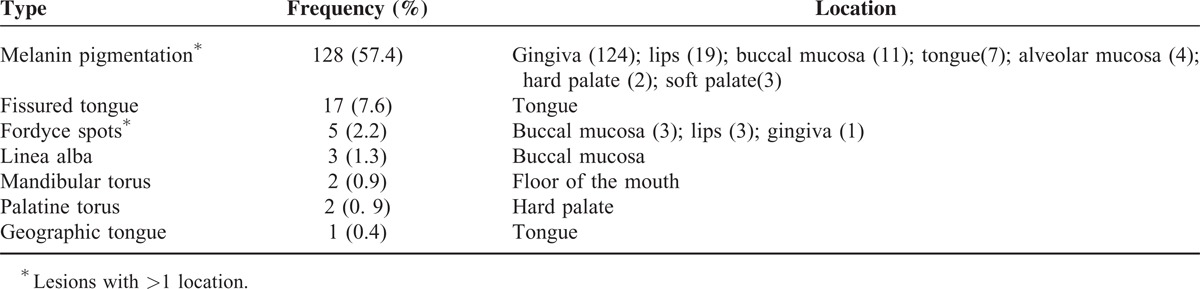
Prevalence of Variations of Normality Among Adult Kiriri Indians (n = 223), State of Bahia, Brazil, 2011

**TABLE 4:**
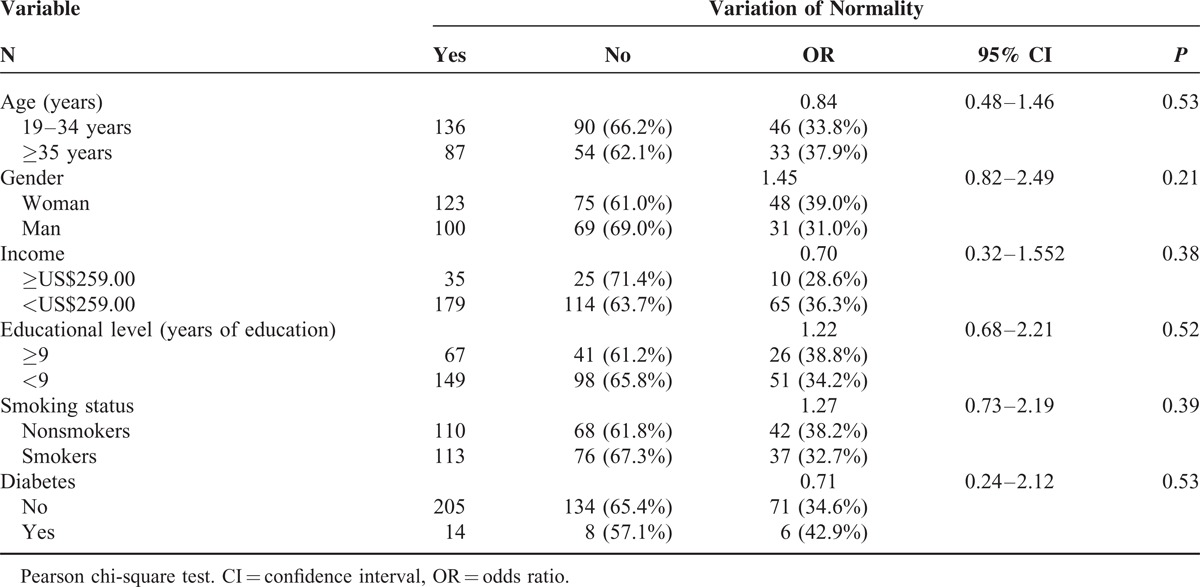
Bivariate Analysis of the Association Between Age, Gender, Income, Educational Level, Smoking Status and Diabetes and the Occurrence of Variations of Normality (N = 223), State of Bahia, Brazil, 2011

## DISCUSSION

In the present study, OMLs were prevalent in 22.4% of the population. Furthermore, older participants were at a significantly higher risk of having OMLs than younger participants, and individuals with a lower educational level were at a higher risk than those with a higher educational level.

In this study, OMLs were found in 50 Indians (22.4%). The prevalence of OMLs has been shown to vary between populations: 9.7% in Malaysia,^4^ 11.8% in the German adult population,^13^ 16.8% in a population from India,^14^ 27.9% in the American adult population,^15^ 53% in elderly people from Chile,^16^ 81.3% in Italian men,^17^ and 22.6% in a Turkish population.^18^ In Brazilian Indians from other communities, a prevalence of between 4.4% and 45.4% has been described.^6,7^ These differences may be due to differences between the populations studied or discrepancies in methodology.

Fistula was the most prevalent type of lesion in Kiriri Indians (6.27%); however, a lower prevalence (0.3%) has been observed in another Indian population.^6^ This indicates that a high proportion of the population has teeth with untreated caries lesions in the Kiriri population, showing a clear need for dental intervention. In fact, the mean number of decayed teeth per person was 4.21.^11^ In other Indian populations, the most prevalent lesions were focal epithelial hyperplasia and candidiasis.^6,7^ In non-Indians, traumatic ulcer,^19^ denture stomatitis,^20,21^ snuff dipper's lesion,^14^ smoker's palate,^22^ and frictional lesion^23^ were the most prevalent lesions. This variability is likely to be related to differences in behaviors and traditions between these populations, as well as the access to dental service.

Traumatic ulcer was the second-most prevalent lesion in the population studied (4.4%), similar to the 6% prevalence found among the Waimiri Atroari community and others.^6,24,25^ However, a higher prevalence of traumatic ulcer (21.5%) has been described in socioeconomically deprived Brazilian population,^19^ and only 0.9% prevalence in a Turkish population.^26^ Traumatic ulcer may be produced by different agents such as bites, altered teeth, apparatus, brushing, maladapted partial or complete removable dentures, malocclusion, dental caries, and unsatisfactory restoration. Therefore, the presence of these agents explains the prevalence differences in the studies. In the Kiriri population, traumatic agents were removed whenever present and the lesions were followed to confirm the diagnosis.

In this population, 4 cases (1.8%) of actinic cheilitis were diagnosed. In Indians, 0.3% and 1.7% prevalence of actinic cheilitis were found.^6,7^ Actinic cheilitis is caused by chronic and excessive exposure to ultraviolet radiation in sunlight. A higher prevalence of actinic cheilitis was expected, as sunlight exposure is intense for Indian populations, and Kiriri Indians live in a tropical climate with a very high intensity of ultraviolet radiation. In a population of workers on sugarcane plantations in Brazil, a prevalence of 39.6% was observed.^27^ However, the melanin skin pigmentation, typical of the Brazilian Indians, may represent a significant protection factor for this lesion.

The prevalence of candidiasis was 2.2%, most of them (n = 3) on the hard palate, and 2 were observed in smokers. Candidiasis are fungal lesions that may be associated with smoking, use of dentures, vitamin deficiencies, high carbohydrate diets, decreased immunity, endocrine disorders, soft tissue lesions, poor oral hygiene, and long-term use of antibiotics and hormones, among others.^28^ In Indians, the prevalence of candidiasis has previously been found to be between 1% and 3.7% and has been shown to be associated with the use of prosthetic appliances.^6,7^ In other populations, prevalence of candidiasis between 0.04% and 2.5% has been reported.^19,22,26^

The prevalence of melanocytic nevus was 2.6%. All detected melanocytic nevi were located on the lips. Among the Waimiri Atroari Indians, melanocytic nevus occurred only in the perioral region, on the skin, and in continuity with red lip, and showed a prevalence of 0.9%.^6^ Epidemiological data on the prevalence of that lesion is scarce. Its development seems to be related to some factors such as skin type, ethnicity, genetic predisposition, and exposure to ultraviolet light.

In the present population, older participants were at a higher risk of OML, which is in agreement with the literature.^5,18^ This finding may be related to the aging process, metabolic changes, nutritional factors, medications, or prosthetic use. In other Indian populations, the prevalence of OMLs was not influenced by age, gender, tobacco use, or diet.^6,7^ Similar to these findings, in the present population, gender, income, smoking status, and diabetes were not found to be risk factors for OML. Although 50% of the population comprised smokers, the mean number of cigarettes smoked per day was 3.7, which may explain the lack of association between smoking status and OML. The present population reported the use of cigarettes (manufactured and hand-rolled with different products), pipes, and a combination of both. In addition, many individuals claimed to smoke only during Indian daily or weekly ceremonies.

Variations from normal mucosa were identified in 144 (64.60%) adult Indians. In accordance with other studies in Indian populations, melanin pigmentation, fissured tongue, linea alba, Fordyce spots, palatine and mandibular torus, and geographic tongue were identified.^6,7^ The identification of variations of normality is important, as a biopsy may be required to establish the correct diagnosis and thereby exclude other lesions.

Physiologic pigmentation of the oral mucosa shows variable prevalence in different ethnic groups. Attached gingiva is the most common intraoral site of such pigmentation, where it appears as a bilateral, well demarcated, ribbon-like, dark brown band that usually spares the marginal gingiva.^29^ This was the most prevalent variation of normality encountered among the Kiriri Indians in this study, and in 124 of the 128 cases, melanin pigmentation was noted in the gingiva.

In the Kiriri population, fissured tongue was the second most common variation of normality (7.6%). In contrast, a higher prevalence of fissured tongue was found among Waimiri Atroari Indians (27.3%).^6^ This variability is probably related to differences in the race, gender, and age of samples and the use of different diagnostic criteria.

The sample size may be a limitation of this study. However, considering the logistical, financial, and political constraints involved in the development of this study, a sampling error of 5% was employed, and no correction factor was used for sample size calculation.

In conclusion, oral mucosal lesions were prevalent in Kiriri Indians, and were associated with an older age and lower education level. A public health program aimed at preventing and treating mucosal lesions targeting the high-risk group is vital to improve the oral health status in this population.
